# Unique neurobiology during the sensitive period for attachment produces distinctive infant trauma processing

**DOI:** 10.3402/ejpt.v7.31276

**Published:** 2016-11-08

**Authors:** Maya Opendak, Regina M. Sullivan

**Affiliations:** 1 Emotional Brain Institute, Nathan Kline Institute for Psychiatric Research, Orangeburg, NY, USA; 2 Child Study Center, Child & Adolescent Psychiatry, New York University School of Medicine, New York, NY, USA

**Keywords:** Trauma, attachment, amygdala, development, stress, sensitive period, infant, maternal care

## Abstract

**Background:**

Trauma has neurobehavioral effects when experienced at any stage of development, but trauma experienced in early life has unique neurobehavioral outcomes related to later life psychiatric sequelae. Recent evidence has further highlighted the context of infant trauma as a critical variable in determining its immediate and enduring consequences. Trauma experienced from an attachment figure, such as occurs in cases of caregiver child maltreatment, is particularly detrimental.

**Methods:**

Using data primarily from rodent models, we review the literature on the interaction between trauma and attachment in early life, which highlights the role of the caregiver’s presence in engagement of attachment brain circuitry and suppressing threat processing by the amygdala. We then consider how trauma with and without the caregiver produces long-term changes in emotionality and behavior, and suggest that these experiences initiate distinct pathways to pathology.

**Results:**

Together these data suggest that infant trauma processing and its enduring effects are impacted by both the immaturity of brain areas for processing trauma and the unique functioning of the early-life brain, which is biased toward processing information within the attachment circuitry.

**Conclusion:**

An understanding of developmental differences in trauma processing as well as the critical role of the caregiver in further altering early life brain processing of trauma is important for developing age-relevant treatment and interventions.

**Highlights of this article:**

- Trauma experienced in early life has been linked with life-long outcomes for mental health through a mechanism that remains unclear.
- Trauma experienced in the presence of a caregiver has unique consequences.
- The infant brain is predisposed toward processing information using attachment circuitry rather than threat circuitry.
- Data from rodent models suggest that repeated trauma in the presence of a caregiver prematurely engages brain areas important for threat, which may play a role in deleterious outcome.

Since the 1950s, we have known that the brains of altricial species, such as humans and rodents, continue to develop after birth, and that experience interacts with genetics to guide the complex process of building the brain (Andersen & Teicher, 2008; De Bellis & Thomas, 2003; Landers & Sullivan, 2012; Levine, 2005; Plotsky et al., 2005; Roth, Zoladz, Sweatt, & Diamond, 2011; Sanchez, Ladd, & Plotsky, 2001). This open system has profound effects on cognitive and emotional development because the brain can be molded to more closely fit diverse environments to enhance survival. However, the plasticity of this system can also be dangerous, as experiences during early life have a heightened saliency. Environmental perturbations, especially if they are extreme or aberrant, can disrupt normal developmental trajectories or initiate a developmental pathway that will result in maladaptive cognitive or emotional behavior or more extreme pathologies, such as mental illness. Understanding this process of environmental influences on brain development has been challenging, not only because the process of building a brain is complex, but because many of the effects of early life trauma are not expressed until a later stage of development or are revealed only in certain circumstances, such as during periods of high stress (Ainsworth, 1969; Gunnar, Quevedo, & Ronald De Kloet, 2007; Landers & Sullivan, 2012; Raineki, Cortés, Belnoue, & Sullivan, 2012). Although our understanding of mechanisms mediating these early-life environmental effects is limited, the quality of caregiving has been identified as a critical variable in mediating the effects of early life stress.

A rich literature has demonstrated the importance of the relationship between a child and a caregiver across a variety of species, a relationship primarily understood in terms of attachment. The importance of attachment in defining cognitive and emotional development was seen in the early works of researchers focusing on both humans and other animals, including, but not limited to, Konrad Lorenz, Niko Tinbergen, Harry Harlow, John Bowlby, and Mary Ainsworth (Ainsworth, 1969; Bowlby, 1978; Harlow & Harlow, 1965; Harmon, Morgan, & Glicken, 1984; Hess, 1962). Through integration of human and animal research, the importance of attachment to the caregiver and the detrimental effects of prolonged separation from the caregiver were identified (Gunnar et al., 2007). Research on rodents and non-human primates quickly identified the hypothalamic-pituitary-adrenal (HPA) axis as one mediator for disrupting development (Gunnar et al., 2007; Rincón-Cortés & Sullivan, 2014; Sanchez, 2006), although other factors are also likely important (Sullivan & Dufresne, 2006). This stress system mediates the body’s response to allostatic load; chronic over-activation of the HPA axis in response to early life trauma can produce long-term adaptations in the HPA response to stress, and these changes are thought to be involved in the pathogenesis of disorders such as posttraumatic stress disorder, depression, and anxiety (Graham, Heim, Goodman, Miller, & Nemeroff, 1999).

## Trauma processing in early life is different: Some brain areas that process adult trauma are not yet mature

The substrate of early life experience on the brain involves changes at nearly every level of analysis, from cellular signaling to behavioral expression. Indeed, through the decades, almost every neurotransmitter system and a multitude of brain regions have been implicated as mediating or impacted by early life experiences, including changes in receptors, neurotransmitter levels, brain structure, epigenetics, the microbiome, immune system, and homeostasis maintenance (Andersen & Teicher, 2008; Bale, 2015; Blakemore & Mills, 2014; Buran et al., 2014; Coria-Avila et al., 2014; Drury et al., 2012; Hartley & Lee, 2015; Heim & Binder, 2012; Knudsen, 2004; Nelson, Lau, & Jarcho, 2014; Pechtel, Lyons-Ruth, Anderson, & Teicher, 2014; Penhune & De Villers-Sidani, 2014; Poulos et al., 2014; Tost, Champagne, & Meyer-Lindenberg, 2015; Umemori, Winkel, Castren, & Karpova, 2015; Werker & Hensch, 2015; Yu et al., 2014; Zannas & Binder, 2014). How these neural changes interface with behavior during development is also complex, as it involves brain areas that functionally develop at different times and scaffolding processes that may be permanently altered.

It is beyond the scope of this review to describe brain development in detail, with excellent reviews available (Casey, Tottenham, Liston, & Durston, 2005; Houston, Herting, & Sowell, 2014). However, a few basic concepts are helpful for the present discussion, which can apply to human, primate, and rodent models. First, the brain develops throughout early life and adolescence, with different brain areas each having their own developmental trajectory and maturation; these developmental trajectories can involve changes in neural connectivity, receptor function, and structure (Berdel, Morys, & Maciejewska, 1997; Brummelte & Teuchert-Noodt, 2006; Chareyron, Lavenex, Amaral, & Lavenex, 2012; Chareyron, Lavenex, & Lavenex, 2012; Cunningham, Bhattacharyya, & Benes, 2002; Ehrlich, Ryan, & Rainnie, 2012; Jagalska-Majewska et al., 2003; Knudsen, 2004; Van Eden & Uylings, 2004; Wakefield & Levine, 1985). Furthermore, all brain areas are involved in myriad behaviors, and each brain area’s microcircuits supporting each behavior have their own developmental trajectory; this makes it nearly impossible to say a particular brain area becomes functional at a specific age. Brain areas involved in basic physiological functions are certainly mature at birth, but continue to mature and develop more complex connections throughout development. Areas involved in complex behaviors and higher order functioning are more delayed in maturation, including the amygdala, hippocampus, and prefrontal cortex (PFC), although emerging evidence suggests that specific functions of each of these brain areas emerge at different ages. In the rodent hippocampus, for instance, long-term potentiation (LTP), a presumed measure of plasticity, emerges during the second postnatal (PN) week (Bekenstein & Lothman, 1991; Harris & Teyler, 1983; Swann, Smith, & Brady, 1990; Wilson, 1984), whereas contextual learning comes online around PN day 23 (Raineki, Holman et al., 2010). Furthermore, some brain areas likely encode information at one age but influence behavioral expression at a later age (Pattwell et al., 2012; Poulos et al., 2014). Emerging evidence also suggests brain areas can have unique function in early life, such as the important role of the rodent’s locus coeruleus (LC) norepinephrine (NE) in attachment that is described below (Landers & Sullivan, 2012). Finally, the numerous connections between brain areas can be further delayed in maturation so that feedback systems that form complex loops of bidirectional information processing occur at a later age. How these complex circuits develop in humans is not fully understood. Thus, as we consider how early life trauma can have an impact on the child, it is important to consider whether brain areas implicated in adult trauma processing are available from an early age.

Several key brain areas that are important for processing trauma and threat are the amygdala, hippocampus, and PFC, and, as noted above, maturation of these brain areas and their connectivity occurs slowly over early life in humans (Bachevalier & Loveland, 2006; Casey et al., 2005; Gee et al., 2013; Graham et al., 1999; Holland & Gallagher, 2004; Lavenex & Banta Lavenex, 2013; Malter Cohen et al., 2013; Sanchez et al., 2001; Skuse, Morris, & Lawrence, 2003; Tottenham, 2012; Tottenham & Sheridan, 2009), non-human primates (Bachevalier & Loveland, 2006; Graham et al., 1999; Holland & Gallagher, 2004; Lavenex & Banta Lavenex, 2013; Sanchez et al., 2001; Skuse et al., 2003) and rodents (Brummelte & Teuchert-Noodt, 2006; Gee et al., 2013; Graham et al., 1999; Holland & Gallagher, 2004). The amygdala is considered to be the critical structure involved in the formation and storage of conditioned fear associations (Davis, Rainnie, & Cassell, 1994; Phelps & LeDoux, 2005), with the lateral nucleus acting as the site of storage and plasticity for fear memories and the central nucleus acting as the fear output center through its projections to downstream structures involved in expression of fear (e.g., paraventricular nucleus) (Krettek & Price, 1978; LeDoux, Iwata, Cicchetti, & Reis, 1988; Phelps & LeDoux, 2005). The amygdala also has a role in many emotional systems, including those unrelated to fear, such as social odor processing and assessing hedonic value of stimuli (Holland & Gallagher, 2004; Maren & Fanselow, 1996; Phelps & LeDoux, 2005; Royet et al., 2000). The basic neuroanatomical architecture of the human amygdala is present by birth (Humphrey, 1968; Ulfig, Setzer, & Bohl, 2003) and in females, amygdala growth is complete by 4 years old (Giedd et al., 1996). Using the visual cue of fearful faces, it has been suggested that amygdala function emerges around 6–7 months of age in human infants (Jessen & Grossmann, 2015; Jones, Laurens, Herba, Barker, & Viding, 2009). This assessment is concordant with behavioral measures of amygdala emergence in children, including separation anxiety and fear of heights (Jessen & Grossmann, 2015; Jones et al., 2009; Strawn et al., 2014, 2015). Once this late developing brain area is functional, it plays a strong role in learning about threat and other behavior.

Evidence from human studies suggests that functional connectivity between the amygdala and hippocampus appears to be delayed, although this connectivity is important during threat processing in adulthood. The hippocampus is a system with diverse function, including the ability to remember specific information about events, such as where and when events occurred. Because the child’s hippocampus is difficult to image using brain scanning techniques, there is no data on hippocampal functional emergence, although hippocampal growth rates greatly slow down as a child approaches 2 years old (Qin et al., 2014). Evidence from rodent studies on development of the amygdala and hippocampal development show a similar developmental time course; both regions demonstrate considerable growth during the initial PN period, coming online at PN10 and PN23 in normal rearing conditions (Chareyron, Lavenex, Amaral, et al., 2012; Chareyron, Lavenex, & Lavenex et al., 2012; Raineki, Holman et al., 2010). Finally, the specific developmental trajectory of the PFC remains incompletely understood. For example, evidence from human fMRI studies suggests that the PFC subarea orbitofrontal cortex, which has an important role in processing the hedonic value of odors (Anderson et al., 2003; Gottfried, Deichmann, Winston, & Dolan, 2002; Rolls, 2015; Zald & Pardo, 1997) is postulated as functional by the time the child is 2 or 3 years old, whereas the anterior cingulate cortex and medial PFC are thought to possibly come online around 4 months and as early as 4 years, respectively (Allman, Hakeem, Erwin, Nimchinsky, & Hof, 2001; Gee et al., 2013; Graham et al., 2015). Although these structures are robustly involved in trauma processing during adulthood, their involvement in processing early-life trauma is complex, because of their limited maturity and/or functional unavailability. Research on animal models has added a new layer of complexity to this work by showing that traumatic experience itself can affect the developmental trajectory of brain areas involved in trauma processing.

## Infant trauma processing: Importance of the caregiver as regulator of brain and behavior

The ability to self-regulate emotional and physiological responses is a critical factor for processing trauma. This self-regulatory system gradually develops with maturation and the young child typically has access to a dependable caregiver who has the ability to regulate the child until his or her independent system is available. This caregiver regulation of infant’s physiology has been shown to be critical for a child’s response to trauma but also interaction with the world, such as response to novelty (Nachmias, Gunnar, Mangelsdorf, Parritz, & Buss, 1996). Caregiver regulation of the infant is seen during perfunctory caregiving, as the emotional state is altered by smiling or tickling a baby to increase arousal, or by soothing a crying infant. This stimulation of a baby’s sensory systems changes the baby’s physiology; for example, soothing a stressed baby can lower stress hormone levels as well as regulate the baby’s brain. This concept of the caregiver as a regulator of infant physiology and brain was first presented in the 1980s by Myron Hofer, who identified the mother as a “hidden regulator” of infant behavior and physiology (Hofer, 1984, 1994). Using a rodent model, he and his colleagues identified critical maternal behaviors—focusing on how a specific maternal behavior activated a specific sensory system in a specific pattern and intensity to control basic physiological functions, such as heart rate, activity level, but also brain neurotransmitters, such as NE (Stone, Bonnet, & Hofer, 1976). Although many different people can help regulate the baby, the caregiver gains special access to this regulation as the baby learns about the caregiver from birth.

The literature suggests that the quality of care an infant receives from the caregiver alters how well the caregiver can regulate the infant, as well as the type of attachment the infant will form. Although there are natural variations in maternal care, extremely stressful circumstances can push these normal variations into abusive, neglectful, and trauma-associated maternal care; these can engender a disordered attachment, which is associated with a decreased regulatory capability in the caregiver (Carlson, Hostinar, Mliner, & Gunnar, 2014; Hostinar, Sullivan, & Gunnar, 2014). In humans, disordered attachment related to trauma is linked with social behavioral problems in later life (Bryant, 2016; Caron, Weston-Lee, Haggerty, & Dozier, 2016) and there is some evidence for a link with depressive symptoms (Shevlin, Boyda, Elklit, & Murphy, 2014; but see Fearon, Bakermans-Kranenburg, Van Ijzendoorn, Lapsley, and Roisman, (2010); however, the mechanisms for these changes remain poorly understood.

As mentioned above, the effects of trauma on the brain are ubiquitous, but the link between trauma and lifelong behavioral impairments highlights the importance of the amygdala, hippocampus, and PFC. Although these brain areas serve complex and diverse functions, threat processing has been a major focus because of its link to trauma and aberrant detection of threat, vigilance, and regulation of emotion in mental illness in children and adults (Jedd et al., 2015; Tottenham & Sheridan, 2009). We rely on animal research to tease apart these correlations and highlight mechanisms, which has shown that trauma in early life produces structural and functional changes in the amygdala and a changed threat system (Bagot et al., 2009; Caldji et al., 1998; Elzinga, Schmahl, Vermetten, Van Dyck, & Bremner, 2003; Ivy, Brunson, Sandman, & Baram, 2008; Maestripieri, Tomaszycki, & Carroll, 1999; Raineki et al., 2012; Roth & Sullivan, 2005; Sanchez et al., 2001; Tang, Reeb-Sutherland, Romeo, & McEwen, 2014; Teicher et al., 2003). By modeling attachment in normal and traumatic circumstances in infant rats, we can explore some of the neural mechanisms by which early life attachment programs cognition and emotional processing throughout the lifespan.

## The infant attachment circuit

The infant brain is not an immature version of the adult brain, but rather, is designed for forming attachments during a temporally constrained sensitive period in early life. Evolution has ensured that the infant brain of altricial species is designed to support attachment to the caregiver, even when the quality of care is compromised or even abusive. This relies upon the unique neurobiology of the altricial infant brain, which processes even traumatic information by the attachment circuitry, rather than the regions used to process trauma in adulthood. A useful framework in which to understand this is that the young brain continuously morphs to accommodate the specific behavioral niche at each developmental stage. For example, the young infant does not need a brain that supports behaviors to gather food from the environment or raise money to support a family; rather, the brain is designed to engage the caregiver to provide those resources needed for survival (Spear & Rudy, 1991). The child gains different adult-like functioning for different tasks at different ages and this is presumably supported by transitions in brain function and development.

A robust literature posits a unique learning circuit involved in the formation of attachment across a variety of species, although the neurobiology of infant attachment has mostly been described using rodents. Similar to imprinting in avian species wherein the chick learns to express prosocial behaviors to the caregiver, rodents also use a unique learning circuit within the brain to support attachment to the caregiver. This circuit involves the olfactory system, the piriform cortex, and the LC, as will be discussed below. Infant rodents, called pups, make an excellent model for understanding attachment learning because, similarly to humans and other primates, they must learn to attach to the caregiver, which can be a male or female. Pups can neither see nor hear until the third week of life, and olfaction is the main sensory system used for interactions with the caregiver (newborn humans use all of their sensory systems (Ehret, 1976; Weber & Olsson, 2008). Specifically, maternal odor is of paramount importance to survival, as pups rely on this cue for proximity seeking, nipple attachment, and social behavior; without these, pups cannot access nourishment, thermoregulation, or maternal care. Indeed, anosmic pups rarely survive and, without the maternal odor, pups frequently fail to nipple attach and can become malnourished (Sullivan, Wilson, Wong, Correa, & Leon, 1990).

Similarly to assessment of human behaviors at birth, the incredibly complex dance of caregiver-infant interaction was considered to be innate. However, careful research indicates that learning is of major importance for activating behavioral systems that are age-relevant and biologically predisposed. Specifically, pup approach behavior toward the maternal odor was considered to be guided by pheromones; we now know that the maternal odor is rapidly learned (Leon, 1992; Sullivan & Leon, 1986; Sullivan & Wilson, 1991). This learning process begins in the prenatal environment, where amniotic odors acquire a preferred valence and direct nipple attachment as soon as pups are born. The odor itself is arbitrary, as neutral odor can acquire the value of a maternal odor if placed into the amniotic fluid a few days before birth (Hepper & Cleland, 1998; Pedersen & Blass, 1982; Smotherman & Robinson, 1987). Outside of the womb, a new maternal odor can be rapidly learned; this is especially important to ensure a robust attachment, given the fact that a dam’s odor can change with her diet. For example, a novel odor (e.g., peppermint) placed either on or in the vicinity of the mother will readily take on the properties of maternal odor (Cheslock, Varlinskaya, Petrov, & Spear, 2000; Roth & Sullivan, 2005; Sullivan et al., 1990). Outside the nest, if a novel odor paired with a stimulus that mimics maternal behavior, such as milk, warmth, or stroking (grooming), this odor acquires the value of a new maternal odor that is not only preferred, but can support nipple attachment and social behavior in the absence of a natural maternal odor (Roth et al., 2013; Roth & Sullivan, 2005, 2006; Sullivan, Hofer & Brake, 1986). During the first 9 days of life, pups readily learn a new maternal odor through a remarkably simple neurobiological substrate. At this age, learning-associated odor plasticity occurs within the olfactory bulb, the first relay station for olfactory processing. This requires that the odor is paired with copious amounts of NE (Sullivan, Stackenwalt, Nasr, Lemon, & Wilson, 2000; Sullivan, Zyzak, Skierkowski, & Wilson, 1992; Yuan, Harley, Bruce, Darby-King, & McLean, 2000). The LC is the sole source of NE to the olfactory bulb and its unique physiology during early life is crucial to neonatal odor approach learning. The infant LC fails to show habituation or show auto-inhibition to turn itself off (as occurs in older pups and adults), producing the large amounts of NE required for this attachment-related plasticity (Nakamura, Kimura, & Sakaguchi, 1987; Winzer-Serhan, Raymon, Broide, Chen, & Leslie, 1996). The olfactory bulb exhibits enhanced responding to the learned maternal odor, as determined by a host of anatomical and physiological changes (Raineki, Shionoya, Sander, & Sullivan, 2009; Roth & Sullivan, 2006; Sullivan et al., 1990; Yuan, Harley, McLean, & Knöpfel, 2002). The olfactory bulb axons of mitral cells project directly to the piriform cortex (Haberly, 2001; Schwob & Price, 1984; Swanson & Petrovich, 1998; Wilson & Stevenson, 2003); this region plays an important role in assigning the hedonic value to a learned odor in a region-specific manner. In particular, the anterior piriform is engaged by odors learned during this sensitive period, whereas the posterior piriform is engaged in response to learned odor aversions in older pups and adults (Moriceau & Sullivan, 2006; Moriceau, Wilson, Levine, & Sullivan, 2006; Roth & Sullivan, 2005). It is important to note that both natural maternal odor and a learned artificial maternal odor generate the same responses from the olfactory bulb (Raineki, Pickenhagen et al., 2010; Roth & Sullivan, 2005). The sensitive period ends when pups are around 10 days old, as the LC becomes more adult-like, where NE release is greatly restricted and specific. At this point, NE takes on a modulatory role in odor learning that is more similar to what has been described in adult rats (Ferry & McGaugh, 2000).

A similar learning about the caregiver cues occurs in humans (DeCasper & Fifer, 1980; Sullivan, Perry, Sloan, Kleinhaus, & Burtchen, 2011), which enables an infant to attach to adoptive parents of either sex. Although we are unsure if the mechanism supporting this learning in human infants is the same as the rodent, NE plays a critical role in bond formation in myriad species (Nelson & Panksepp, 1998; Numan & Young, 2016). Furthermore, NE levels are very high at birth and over the first 2 years of life (Lagercrantz & Bistoletti, 1977), suggesting it is a phylogenetically conserved system.

## Trauma processing in early life is different: the attachment circuit

In species throughout the animal kingdom, including humans, young form attachment to abusive caregivers (Harlow & Harlow, 1965; Hess, 1962; Rajecki, Lamb, & Obmascher, 1978; Stanley, 1962). As mentioned previously, we suggest that evolution has ensured that attachments in altricial species are formed to a caregiver regardless of the quality of care provided. Attachments to an abusive or negligent caregiver may therefore have short-term advantages, for example there is neonatal access to care, but long-term consequences associated with compromised threat processing and emotion expression. Furthermore, low quality or inconsistent caregiving can be reflected in the quality of attachments, with abusive or disordered attachments associated with impaired social and emotional behavior throughout the lifespan (Gunnar, Brodersen, Nachmias, Buss, & Rigatuso, 1996; Nachmias et al., 1996). As will be discussed below, these attachments form because of unique infant brain circuitry; however, chronic pairing of caregiving with traumatic cues can produce latent changes to brain areas involved in trauma processing to engender life-long emotional impairments.

The rodent literature provides some clues to understanding the unique response of the infant brain to traumatic cues. In adulthood, rats typically engage the amygdala, hippocampus, and PFC when learning about threatening stimuli. In infancy, immaturity of these brain areas and a brain biased toward forming attachments prevent the acquisition of aversion to a caregiver during a sensitive window. This process of learning to attach to an abusive caregiver occurs in pups within the nest and can be modeled in the laboratory. As will be discussed below, we can induce abusive behavior in a dam by providing her with insufficient nest-building materials; as a result, the dam will exhibit behaviors such as stepping on pups, dragging them across the cage floor and transporting them inappropriately, inducing pain-related vocalizations. Pairing this painful maternal care with a novel peppermint odor does not activate the important survival circuit within the brain to support aversion learning that involves the amygdala. Rather, pups not only learn to approach this odor, but this odor takes on the qualities of maternal odor to support nipple attachment and prosocial behaviors (Roth & Sullivan, 2005; Sullivan et al., 1986).

Although demonstrating that an abusive mother rat can induce attachment in infant rats is important to show cross-species validity, this complex situation makes it difficult to go beyond correlations when assessing brain mechanisms responsible for this paradoxical pain-associated attachment. For that reason, we use a very precise situation that permits experimental control with an eye toward identifying causal mechanisms. Because attachment is learned and pups rely on the olfactory system for attachment, we use an olfactory classical conditioning learning paradigm in neonatal rodents. This approach is similar to what had been used previously by other labs to determine how pups learn the maternal odor. Specifically, just as new maternal odors can be learned when a previously neutral odor is paired with stimuli evoking maternal care, such as milk or stroking, we paired an odor with a moderately painful foot-shock (0.5 mA) or tail-pinch. On a behavioral level, we knew that pairing moderate pain would produce an odor preference in young pups (Camp & Rudy, 1988; Haroutunian & Campbell, 1979; Spear, 1978; Sullivan et al., 1986); we later showed this procedure also produces a new maternal odor (Raineki et al., 2012). We then asked, what is different about the infant brain that permits pain-odor pairings to support attachment and not activate survival-dependent amygdala circuits that enable us to learn to avoid painful situations?

The inability of the paired odor-pain procedure to produce fear learning is not related to pups’ inability to detect the aversive stimulus or feel pain. Noxious stimuli readily elicit <PN9 pup escape responses and the pain threshold does not appear to change as shock switches from supporting preference to supporting aversion learning (Barr, 1995; Collier & Bolles, 1980; Emerich, Scalzo, Enters, Spear, & Spear, 1985; Stehouwer & Campbell, 1978). As described above, the pup’s olfactory bulb, anterior piriform cortex, and a hyperfunctioning LC work together to generate enhanced odor preference learning to produce an attachment. Furthermore, as will be described in the next section, a hypofunctioning amygdala and blunted levels of circulating corticosterone (CORT) in an immature stress system appear to explain why pups learn to attach rather than avoid caregivers associated with pain (Barr et al., 2009; Moriceau et al., 2006; Sullivan, Landers, Yeaman, & Wilson, 2000).

This combined approach of using an ecologically-relevant model of maternal abuse as a result of low bedding resources, and experimentally controllable situation of odor-shock proxy abuse outside the nest provides unique insight and better understanding of why abuse-related attachments are formed and maintained. Indeed, this abuse-related attachment appears phylogenetically conserved and occurs across species, including chicks that form attachments after being shocked during imprinting (Hess, 1962; Rajecki et al., 1978), dogs (Stanley, 1962), and monkeys raised with a wire surrogate that inflicts pain (Harlow & Harlow, 1965). More recent work has modeled abusive caregiving in non-human primates and again shows that infants retain strong preference for the abusive caregiver (Maestripieri et al., 1999; O’Connor & Cameron, 2006; Sanchez et al., 2001; Suomi, 2003).

In most species, stress and low resources are believed to be major causes for the abuse and neglect of offspring. As mentioned above, this can be modeled in rats by limiting the dam’s nest-building material. When resources are constrained in this manner, the dam exhibits frequent nest building, rough handling of pups, decreased nursing, and trampling of pups (though normal weight gain occurs) (Raineki, Moriceau et al., 2010; Roth & Sullivan, 2005). Despite this rough handling, pups’ approach toward the mother is unaffected in the low-bedding condition, and if a neutral odor is present during the rough treatment, pups will show a preference for the odor outside of the nest. It is important to note that, in this model, the mother still cares for pups, but this care is tempered with rough treatment. As such, this low bedding model may be even more informative about real-life conditions of abuse-related trauma where typical care is punctuated with bouts of abuse and maturation occurs at a typical rate. These results from our maltreatment low-resources model complement other infant models of early life experience that range from low care within a normal range of maternal care (Meaney, 2001), to maternal separation and novelty (Callaghan & Richardson, 2014; Plotsky et al., 2005; Tang et al., 2014), to the highly abusive mother produced by more extreme reduction in nest building material combined with an aversive wire mesh floor (Ivy et al., 2008). In the nest, pups receive a breadth of cues from the mother, including not only normal nurturing in the absence of abuse but also varying degrees of maternal care in the form of licking and grooming. Rodent models of maternal responsiveness, including genetically engineered variants in levels of licking and grooming, have been shown to program stress reactivity in rat pups throughout the lifespan (Claessens et al., 2011); these results parallel the importance of attunement and responsivity in human parenting, especially in highly stressful environments (Asok, Bernard, Roth, Rosen, & Dozier, 2013; Caron et al., 2016). Variations in maternal responsivity can be potentiated by environmental conditions; specifically, high levels of anxiety and/or neophobia resulting from low resources can result in low licking and grooming (Caldji et al., 1998). A growing literature has shown that low levels of licking and grooming in dams is correlated with impairments in measures of social behavior and emotionality in pups throughout the lifespan, related to levels of GABA receptor expression in the amygdala (Asok et al., 2013; Caron et al., 2016; Curley & Champagne, 2015). Together, these models and others have shown that the type, intensity, duration, and age of the early life experiences can produce a variety of distinct neurobehavioral phenotypes in later-life, converging on disrupted HPA axis function as a substrate for neurobehavioral impairments.

## Terminating the sensitive period for attachment learning: the role of CORT

As noted above, we have identified a sensitive period for attachment learning in infant rodents when they are primed for odor learning to support acquisition of a maternal odor and approach the caregiver, even if that odor is associated with painful stimuli. Before PN10, attachment circuitry processes odor pairings via the anterior piriform cortex and the olfactory bulb, but not the amygdala. Importantly, pain information reaches the amygdala, but the structure fails to exhibit plasticity normally associated with fear learning (Barr et al., 2009; Ehrlich & Josselyn, 2016; Herry & Johansen, 2014; Thompson, Sullivan, & Wilson, 2008). The failure of amygdala engagement in learning before P10 is not because of an immature amygdala *per se*, but rather because of pups’ naturally low levels of the stress hormone CORT (Moriceau & Sullivan, 2006; Sullivan, Landers et al., 2000). Whereas stressful stimuli engage the HPA axis and potentiate release of CORT in adults, young pups exhibit low CORT levels and fail to mount a stress response in response to shock; they are said to exhibit a “stress hyporesponsive period” (SHRP), which overlaps with the sensitive period for attachment (Grino, Paulmyer-Lacroix, Faudon, Renard, & Anglade, 1994; Henning, 1978; Levine, 1962; Rosenfeld, Suchecki, & Levine, 1992; Walker, Sapolsky, Meaney, Vale, & Rivier, 1986). Typically, the termination of this SHRP begins around PN10, with decreasing levels of NE and rising endogenous levels of CORT, resulting in functional emergence of the amygdala (Moriceau et al., 2006; Sullivan, Landers et al., 2000). Pups’ increase in stress hormone at this age permits the infant amygdala to show learning-induced plasticity, permitting odor-pain pairings to now activate the fear learning circuit and avoid stimuli that signal threat and danger.

While CORT levels naturally increase at PN10, the environment readily changes pups’ CORT to control the duration of the sensitive period for attachment. Specifically, administering CORT in pups during the SHRP precociously engages the amygdala and permits aversion learning. Pups reared by an abusive mother during the SHRP receive CORT through her milk; when these pups are trained on an odor-shock conditioning paradigm outside the nest at PN7, they will learn an aversion through amygdala-dependent mechanisms (Moriceau, Roth, & Sullivan, 2010; Raineki, Moriceau, & Sullivan, 2010). Although the amygdala is prematurely engaged in abused pups, they will nonetheless form a disordered attachment to the mother, expressed in decreased approach behavior toward the maternal odor and less nipple attachment (Raineki, Moriceau et al., 2010). Similarly, pups reared with a normal nurturing mother but trained on a 5-day odor-shock conditioning procedure during the SHRP will learn an aversion to the odor if they receive CORT injections before each training session (Raineki, Moriceau et al., 2010). Similar effects have been observed in a rat model of maternal separation: pups whose mothers were periodically removed from the nest and pups that received CORT injections both showed precocial abilities to retain memory for fearful stimuli (Callaghan, Sullivan, Howell, & Tottenham, 2014). As will be discussed below, premature engagement of the fear-learning circuitry can have deleterious consequences for its function throughout the lifespan (Callaghan et al., 2014; Moriceau, Roth, Okotoghaide, & Sullivan, 2004; Moriceau, Shionoya, Jakubs, & Sullivan, 2009; Moriceau & Sullivan, 2004, 2006).

Although the specific time-course is unknown, there appears to be a similar SHRP in human children. Infants exhibit a period of dampened cortisol reactivity that develops over the first year of life (6–12 months) (Gunnar & Donzella, 2002; Gunnar, Hostinar, Sanchez, Tottenham, & Sullivan, 2015). Although the duration of this period is unknown, basal cortisol levels remain low through the preschool period (Grunau, Weinberg, & Whitfield, 2004; Watamura, Donzella, Kertes, & Gunnar, 2004). Some studies show that trauma experienced prior to age 7 years old (needing to be rescued while swimming) was not associated with fear of swimming when measured at age 18 (Poulton, Menzies, Craske, Langley, & Silva, 1999). In contrast, late-onset (after 18 years old) dental fear, but not early-onset dental fear, was associated with aversive conditioning experiences at the dentist’s office (Poulton, Waldie, Thomson, & Locker, 2001). These studies are consistent with the hypothesis that in typical development, the amygdala-dependent fear system is less engaged during emotional learning than during adulthood. Laboratory-based fear conditioning studies have measured galvanic skin responses and heart rate and with young children show that fear conditioning increases from the preschool period to middle childhood (Gao, Raine, Venables, Dawson, & Mednick, 2010). Further study will be required to determine whether the hyporesponsivity of the stress system results in preference learning in humans as it does in the rodent (Moriceau et al., 2006).

## Post-sensitive period of attachment learning: Social buffering of the CORT response by the mother reinstates the sensitive period

**Fig. 1:**
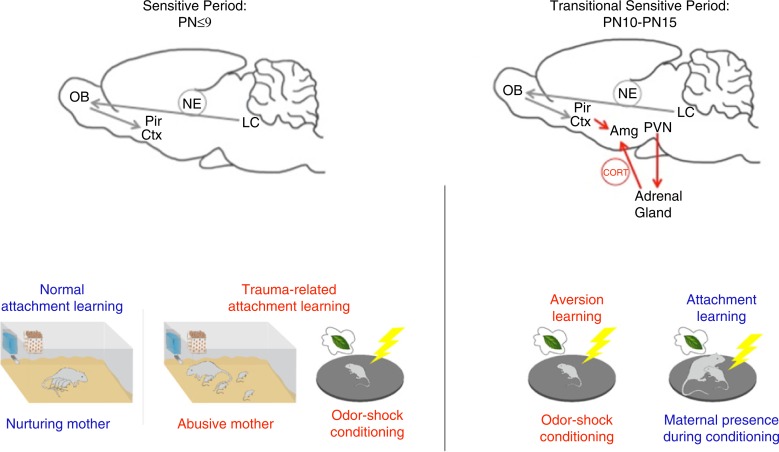
Using a fear conditioning paradigm of odor-shock presentations has enabled us to uncover a developmentally unique learning system in pups that typically supports attachment learning. Data indicate that during the sensitive period for attachment learning (PN≤9), low CORT levels block amygdala plasticity to prevent pups from learning amygdala-dependent fear/threat. Instead, this learning paradigm activates the attachment learning neural circuit involving NE to produce approach responses to the odor (Moriceau et al., 2006). The odor also takes on qualities of the maternal odor to support nipple attachment and enhance prosocial behaviors to the mother. In pups older than PN9, this fear conditioning paradigm accesses the amygdala to support fear/threat learning if the pup is alone. A critical feature of this learning is that shock induces activation of the HPA axis and CORT release, which is necessary for the young amygdala to learn. However, if the mother is present, she socially buffers the pup’s stress response, and pups revert to sensitive period learning and learn an odor preference. This mother-controlled switch between fear and attachment learning is mediated through the mother’s ability to control pups’ CORT (Moriceau, Wilson et al., 2006). A more adult-like fear learning system, which cannot be switched on/off by CORT, develops by PN15. Environmental variables that control pups’ CORT level, such as receiving CORT from a stressed mother via milk, environmental manipulations that increase pups’ CORT (exogenous CORT administration and abusive rearing), or the mother’s ability to socially buffer the pups (compromised in abusive mothers) have the potential to modify the age of these transitions and whether a pup learns fear or attachment (Moriceau & Sullivan, 2006; Perry & Sullivan, 2014; Raineki et al., 2012; Shionoya et al., 2007; Sullivan & Holman, 2010).

In normal conditions, shock begins to increase CORT in PN10 pups and pups readily learn to avoid odors paired with shock, indicating the functional emergence of amygdala-dependent fear learning (Sullivan, Landers et al., 2000). Before PN10, exogenous CORT administration (through a stressed mother’s milk and through micro infusions to the amygdala) prematurely engaged the threat system and produced aversion learning to a conditioned odor; therefore, we wondered whether decreased CORT in >PN10 pups could reinstate the sensitive period for attachment. We first used a controlled pharmacological approach where CORT was artificially lowered either systemically (3 mg/kg) or just within the amygdala (50–100 ng) during the odor-shock proxy abuse procedure, both of which reinstated attachment learning in PN10–15 pups (Moriceau et al., 2006). We next used a nurturing (non-abusive/non-stressed) mother to naturally lower pups’ CORT through social buffering—the process by which a significant social partner can attenuate the stress response (Moriceau & Sullivan, 2006; Shionoya, Moriceau, Bradstock, & Sullivan, 2007); in young pups maternal presence can completely block release of the stress hormone CORT (Stanton & Levine, 1990; Stanton, Wallstrom, & Levine, 1987; Suchecki, Rosenfeld, & Levine, 1993). These studies showed that between PN10 and PN15, the “transitional sensitive period”, maternal presence (non-abusive) can act as a switch to turn off the fear circuitry and turn on the attachment circuitry during odor-shock conditioning simply by blocking CORT (Fig. 1) (Moriceau & Sullivan, 2006). After PN15, however, only fear is learned during odor-shock conditioning, regardless of maternal presence or CORT manipulation (Upton & Sullivan, 2010).

In humans, it is not yet known whether the mother exerts a similar buffering effect over amygdala reactivity. It has been shown, however, that a mother’s presence can buffer against cortisol elevations, particularly for highly inhibited or fearful children (Nachmias et al., 1996). Other studies have shown a key regulatory role for the caregiver in buffering behavioral and neuroendocrine stress responses as well as the acquisition of conditioned fear and inhibitory control before adolescence (for review, see Callaghan & Tottenham, 2016). It follows that low quality caregiving can result in poor regulation in children, resulting in aberrant development of brain areas related to fear and emotion. Indeed, social buffering is compromised in children with disordered attachments to a caregiver (Ainsworth & Bell, 1970; Nachmias et al., 1996). Furthermore, studies of children raised in orphanages abroad, where care is inconsistent or of low quality, have shown a correlation between amygdala volume and age of adoption. These volumetric data correlate with behavioral phenotypes; as a group, previously-institutionalized children are more likely to exhibit heightened anxiety and internalizing problems—markers of increased vigilance (Tottenham, 2012). These results are consistent with the notion that the absence of consistent or high-quality early caregiving alters emotional behavior by acting on amygdala development.

## Uncovering trauma-caregiver effects in early life

**Fig. 2:**
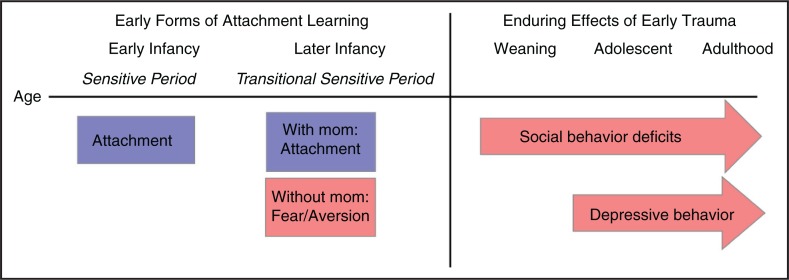
Timeline of attachment learning and the effects of early life maltreatment on later-life social and emotional behavior in the rat model of trauma associated with attachment. Early infants will learn attachment regardless of the quality of care, whereas slightly older infants (PN10–15) will either learn to fear a traumatic associated stimulus when away from the mother or learn an attachment if acquisition takes place with the mother. Testing later in life shows that only the early life trauma associated with attachment will lead to lifelong amygdala-dependent behavioral deficits, such as poor social behavior (onset prior to weaning) and depressive-like behaviors (onset post-weaning (Raineki et al., 2012; Sevelinges et al., 2011; Sullivan et al., 2000)). In pre-weanlings, poor social behavior is defined as decreased nipple attachment and fewer choices toward the maternal odor, indicating disordered attachment. In adolescence and adulthood, rats with a history of abuse will exhibit decreased preference for a social stimulus over a non-social stimulus in Crawley’s three chamber test. This is in sharp contrast to experience with trauma without attachment, which results in later-life anxiety (Rincón-Cortés & Sullivan, 2014; Sarro et al., 2014).

When rat pups are exposed to the odor-shock model of early-life maltreatment from PN3 to PN8, their social behavior toward their mother is normal in measures of nipple-attachment and choices toward her odor in a Y-maze (Raineki et al., 2012; Rincón-Cortés et al., 2015). However, an injection of CORT (modeling a heightened stress environment) in PN8 pups revealed a hyperactive amygdala and strong deficits in social behavior toward the mother (i.e., fewer choices toward a maternal odor and less time nipple-attached (Raineki et al., 2012)). These results parallel data wherein stress uncovered latent neurobehavioral deficits in children with disordered attachment resulting from abuse or neglect (Ainsworth, 1969; Gunnar et al., 1996). CORT injection did not activate the amygdala or disrupt attachment behaviors in pups reared with a control nurturing mother or pups subjected to odor-stroke conditioning. As mentioned above, repeated CORT injection to <PN10 pups precociously ends the SHRP, resulting in activation of the amygdala and producing aversion learning; it is unknown whether this procedure produces long-term changes in amygdala function (Moriceau, Raineki, Holman, Holman, & Sullivan, 2009; Moriceau & Sullivan, 2006; Moriceau et al., 2006). In addition, pups reared with an abusive mother from PN3–8 were shown to have increased CORT, heightened amygdala reactivity, and impaired attachment behaviors with the mother, suggesting that chronic stress induced premature amygdala engagement and terminated the SHRP (Raineki et al., 2010). Importantly, these data from CORT injection studies suggest that maltreatment by an abusive caregiver or experience with repeated pairing of traumatic shock with attachment circuitry changes amygdala development so that stress produces a hyperactive amygdala that halts normal social behavior (Ainsworth, 1969; Gunnar et al., 1996). Furthermore, these stress-induced neurobehavioral deficits in infancy also predicted later life depressive-like behaviors and amygdala dysfunction (Raineki et al., 2012). Specifically, rats with a history of maltreatment exhibited social behavior deficits with peers as early as PN20, marked by decreased exploration of a conspecific in a three-chamber test of sociability (Crawley, 2004). This preceded depressive-like symptoms in sucrose consumption and forced swim tests beginning in adolescence (PN45) and persisting through adulthood, accompanied by a hyperfunctioning amygdala (Fig. 2) (Raineki et al., 2012; Sevelinges et al., 2007, 2011; Sevelinges, Sullivan, Messaoudi, & Mouly, 2008). Temporary inactivation of the amygdala via muscimol infusion normalized behavior, indicating a causal role for the amygdala in these deficits. These results suggest that how the effects of attachment-trauma are expressed changes during development, but implicates amygdala dysfunction at both ages. These results mirror results in humans, where childhood dysfunctional social behavior occurs prior to onset of depression (Letcher, Smart, Sanson, & Toumbourou, 2009; Mason et al., 2004; Mazza, Fleming, Abbott, Haggerty, & Catalano, 2010).

## Enduring impact of infant trauma with the caregiver

Early life trauma produces vulnerability to later life mental health difficulties and psychiatric disorders, as well as compromises cognitive and emotional development. This has been modeled in research involving many animal species since the 1950s (Andersen & Teicher, 2008; Denenberg, 1963; Famularo, Kinscherff, & Fenton, 1992; Harlow & Harlow, 1965; Hofer, 1994; Levine, 1957; Levine, Johnson, & Gonzalez, 1985; Teicher et al., 2003). This is also shown in our models: rearing by an abusive mother or experiencing repeated paired odor-shock (both models activating the attachment circuitry) results in amygdala-PFC deficits, depressive-like behavior, and social behavior deficits that emerge around weaning age as pups transition to independence (Raineki, Cortes et al, 2012; Raineki et al., 2015; Roth, Matt, Chen, & Blaze, 2014; Sevelinges et al., 2007, 2011, 2008).

Importantly, simply experiencing trauma without engagement of the olfactory attachment circuit (olfactory bulb-LC-anterior piriform) does not produce these neurobehavioral deficits: infant experience with just shock alone results in anxiety-like behavior associated with dysfunctional amygdala GABA processing, although it does not produce a hyperactive amygdala or depressive-like behavior (Sarro, Sullivan, & Barr, 2014; Tyler, Moriceau, Sullivan, & Greenwood-Van Meerveld, 2007). Overall, these results indicate that trauma associated with the caregiver produces unique outcomes, with attachment-related trauma producing more ubiquitous and profound neurobehavioral deficits, compared to trauma alone.

Although the effects of early life caregiver abuse are ubiquitous throughout the brain, we have focused on the amygdala in our rodent model and have identified a causal role for the amygdala in attachment-related trauma. Pups with a history of maltreatment following maternal abuse in a low-bedding model as well as the odor-shock proxy abuse paradigm showed amygdala activation when stressed, suggesting that early-life abusive attachment recruited the amygdala despite pups failing to learn the amygdala-dependent fear (Raineki, Moriceau et al., 2010). These results parallel data in abusive caregiving, which alters DNA methylation in the adult rodent amygdala (Roth et al., 2014). Long-term changes to amygdala function following odor-shock conditioning were observed in a task-specific manner: odor paired with pain to produce a learned attachment odor blunted adult fear conditioning and attenuated amygdala neural activity supporting adult odor-fear learning, whereas social behavior and depressive-like behavior were accompanied by amygdala hyperactivity (Sevelinges et al., 2007, 2008). Interestingly, unpaired presentations of the odor and shock stimuli are known to significantly alter gene expression patterns within the amygdala, despite not producing learned preferences (Sarro et al., 2014). These results suggest that trauma in early life may produce latent changes in the amygdala, potentially underlying some of the long-term behavioral effects associated with abuse, including the development of psychiatric disorders.

## Concluding remarks

Evolution has ensured that the young of altricial species spend a considerable amount of time with the caregiver before they mature and reach independence. In rat pups, this is manifested such that infants learn rapid and robust attachment to their caregiver, regardless of the quality of care that they receive, including harsh and painful interactions. This indicates that neural circuitry normally engaged in assessing and learning about threat is absent or suppressed in the presence of an abusive caregiver. Although this may ensure that the pup does not miss out on adequate resources from the caregiver, there may be a trade-off in healthy threat learning trajectories pursuant to this abusive attachment. Converging with clinical literature, work exploring the neurobiology of infant attachment in traumatic conditions has revealed latent functional changes in trauma-processing circuitry that persist into adulthood. These changes primarily involve the amygdala and are expressed in deficits in social behavior, as well as vulnerability to developing symptoms of depression. Taken together, these results highlight the importance of early-life attachment quality on later life stress reactivity, emotional expression, and mental health outcomes, and the critical mediating role of the amygdala in these effects.

Through the use of a two complementary rodent models of early-life abuse, we have shown that when trauma is repeatedly paired with olfactory learning within the sensitive window for attachment, the attachment circuitry (olfactory bulb-LC-piriform cortex) can prematurely engage the immature amygdala, a key player within adult-like threat-processing circuitry (amygdala-HPC-PFC). This pairing can produce latent amygdala hyperactivity, resulting in impaired emotionality and social behavior lasting into adulthood. Although a growing literature has linked the amygdala, attachment quality, and stress axis development in humans, little is known regarding the specific nature of this relationship. No consensus has been reached on the mapping of rodent age onto human age; data on the ontogeny of fear learning in children draws parallels between weaning, the age at which rats prepare for independence outside the nest, and various developmental milestones in children, including entering school, entering adolescence, or leaving home as young adults (Callaghan & Tottenham, 2016; Gee et al., 2013; Gunnar & Donzella, 2002; Poulton et al., 1999; Tottenham, 2012; Tottenham & Sheridan, 2009). Although early life has been shown as a sensitive period for caregiver modulation of children’s stress axis (Gunnar & Donzella, 2002; Nachmias et al., 1996), the neural correlates and temporal constraints of this social buffering remain unknown. Further work will be necessary to determine whether humans engage the same neural circuitry as rodents in forming attachments to a caregiver and whether the child’s brain is predisposed toward forming preferences at this age.

Here we have reviewed a very selective infant experience: the association of trauma with or without attachment that focused on the amygdala as one causal mechanism initiating the pathway to pathology. Our results complement a vast array of other infant experience models, including removing the source of pups’ sensory stimuli by separating them from the mother (maternal separation/deprivation), altering sensory stimuli from the mother such as occurs with breeding of low maternal responsiveness, exposure to trauma or novelty outside the nest, CORT manipulation, and neonatal handling. Taken together, these manipulations have been critically important in identifying programing of the stress system as a point of convergence in producing adult outcomes. They have also identified a vast array of mechanisms mediating the enduring impact of early life trauma, including alterations in most neurotransmitter systems, neuronal sculpting, molecular events, genetics/epigenetics, and systems-level changes. Understanding how the diverse types of infant trauma experiences relate to the myriad disorders expressed across the lifetime is necessary to understand and develop age-appropriate trauma intervention and treatment.
